# Abdominal pain as the presenting symptom of Takayasu arteritis in an adolescent male: A case report

**DOI:** 10.1097/MD.0000000000011326

**Published:** 2018-06-29

**Authors:** Shaobo Yang, Kuiran Dong, Shan Zheng

**Affiliations:** Department of Surgery, Children's Hospital of Fudan University, Shanghai, China.

**Keywords:** abdominal pain, children, Takayasu arteritis

## Abstract

**Rationale:**

Takayasu arteritis (TA) is a chronic granulomatous inflammation affecting the aorta and its main branches. The clinical symptoms are mainly due to arterial claudication and end-organ ischaemia. Abdominal pain is an uncommon manifestation of TA.

**Patient concerns:**

We present a rare case of TA in a 13-year-old boy who first presented with abdominal pain. An emergency aortic stent implantation and aneurysm embolization were performed, and the intra-operative diagnosis was aortic pseudoaneurysm.

**Diagnoses:**

A consultation with the department of rheumatism determined that the diagnosis was Takayasu arteritis according to the medical history, physical examination and auxiliary examination results.

**Interventions:**

The patient was transferred to the department of rheumatism for treatment with prednisolone and cyclophosphamide.

**Outcomes:**

Six months after the initial presentation, he was doing well clinically with no additional vascular involvement, and his blood pressure had been stabilized with oral antihypertensive drugs.

**Lessons:**

More detailed examinations of children with acute abdominal pain should be performed. Abdominal computed tomography (CT) should be administered, peripheral impulses and arterial bruits should be checked, and blood pressure among the four limbs should be monitored to rule out plausible emergencies such as an aneurysm caused by TA.

## 1 Introduction

Takayasu arteritis (TA) is a chronic granulomatous inflammation affecting the aorta and its main branches, causing stenosis, dilatation, and aneurysms of the vessels. It is found mostly in female patients and is more prevalent in Asian and Latin American countries, with an age of onset usually in the range of 10 to 40 years.^[1]^ The etiology is unknown but is believed to be autoimmune. The clinical symptoms are mainly due to arterial claudication and end-organ ischemia. Usually the process of vascular narrowing is insidious, with good collateral vessel formation. Less commonly, the process of vascular occlusion may be rapid enough to cause critical ischemia of affected vascular territories with devastating consequences such as stroke. However, abdominal pain is an uncommon manifestation of TA. This study reports the case of a 13-year-old boy with TA first presenting with abdominal pain.

## 2 Case report

On May 3, 2018, a 13-year-old boy presented to the general surgery outpatient department of our hospital with a history of intermittent right abdominal pain for one week. He had no history of fever or emesis. Physical examination revealed no abdominal distension or tenderness, and muscle tension was not palpated. Abdominal ultrasonography showed that the right lower quadrant and urinary system were normal. No special treatment was performed, and follow-up was recommended. After 5 days, the boy presented to the general surgery emergency department for aggravating abdominal pain with emesis. Physical examination revealed abdominal tenderness in the left upper abdomen; however, muscle tension was not palpated.

**Figure 1:**
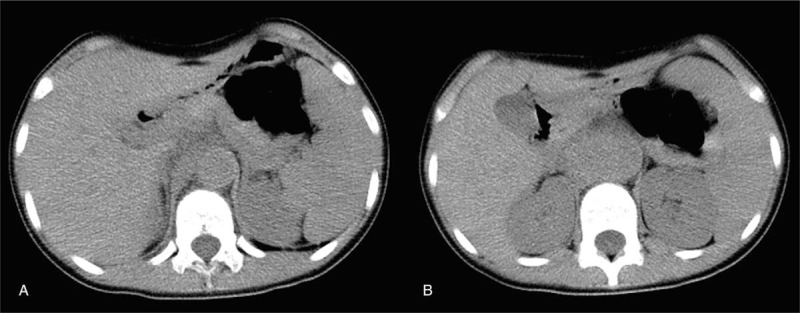
Abdominal CT scan: Enlargement of the upper abdominal aorta, approximately 25.4 mm in diameter (A). The abdominal aorta could not be clarified at the level of the kidney and appeared as mass opacities in the size of 49.6 × 32.3 mm (B).

**Figure 2:**
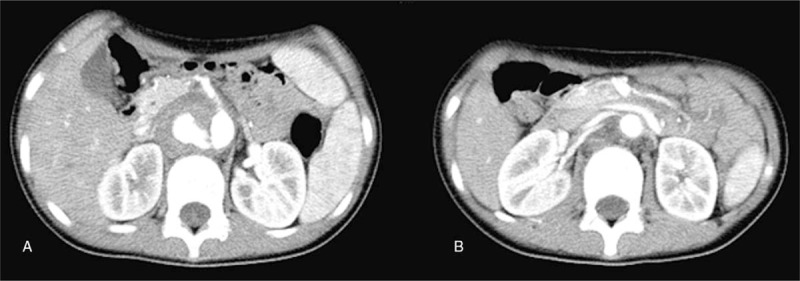
CTA: A crevasse could be seen on the right side of the abdominal aorta at the level of the superior mesenteric artery because of leaking contrast medium (A). Stenosis is seen at the beginning of 2 right-kidney-feeding arteries, which originated from the abdominal aorta and tortuous left-kidney artery (B).

**Figure 3:**
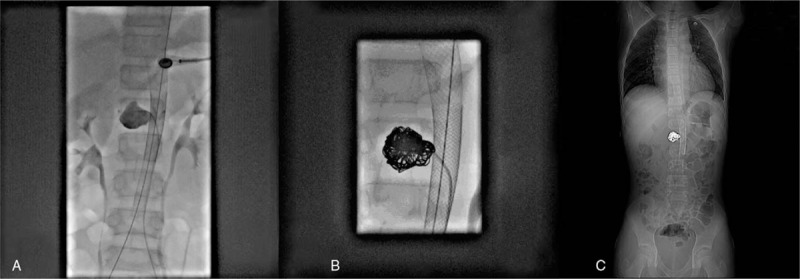
A stent implanted in the aorta and radiography showed the aneurysm (A). Eight spring coils were imbedded (B). The CT scout image showed the stent and spring coils after aneurysm embolization (C).

Routine blood examination showed a white cell count of 14.71 × 10^9^/L (reference range 4–10 × 10^9^/L) with 67.2% neutrophils and C-reactive protein of 59.65 mg/L (reference range < 8 mg/L). An abdominal computed tomography (CT) scan depicted a retroperitoneal occupying lesion in the upper abdomen accompanied by an abdominal aorta dilatation (Fig. 1). The child was then admitted to the department of surgical oncology. On admission, the boy's medical history was found to be unremarkable, and his family history contained nothing of significance. Blood pressure (BP) measurement showed that BP in the right upper limb was 178/94 mm Hg. Laboratory tests revealed the following: white cell count, 9400/μL with 62.8% neutrophils; CRP, 46 mg/L; erythrocyte sedimentation rate (ESR), 23 mm/h (reference range 0–21 mm/h). Computed tomography angiography (CTA) of the aorta showed an aortic pseudoaneurysm (Fig. 2). The boy was transferred to the pediatric intensive care unit (PICU) for further treatment. An urgent consultation was held with the department of vascular surgery of Zhongshan Hospital of Fudan University. An emergency aortic stent implantation and aneurysm embolization were performed the next morning, and the intraoperative diagnosis was aortic pseudoaneurysm (Fig. 3).

After the operation, nicardipine hydrochloride and metoprolol were introduced to lower the patient's BP. A consultation with the department of rheumatism determined the diagnosis to be TA, based on the boy's medical history, physical examination, and auxiliary examination results. The patient was then transferred to the department of rheumatism for treatment on May 9. Treatment with prednisolone was initiated (48 mg daily). Five days after the boy received intravenous prednisolone, cyclophosphamide was administered at a dose of 600 mg. Six months after the initial presentation, he was doing well clinically with no additional vascular involvement, and his BP was stable with oral antihypertensive drugs.

## 3 Discussion

TA is a form of large vessel granulomatous vasculitis characterized by massive intimal fibrosis and vascular narrowing, most commonly affecting the aorta and its branches. Even though infection, inheritance and autoimmunity have been confirmed as important factors during the pathogenic process, the exact cause of TA still remains unknown. Epidemiologically, those with the disease are mostly young women. TA is characterized by segmental and patchy granulomatous inflammation of the aorta and its major derivative branches, which leads to arterial stenosis, thrombosis, and eventually dysfunction of affected organs.^[2,3]^ Reported cases of TA in children are uncommon and mostly have no typical clinical symptoms; hence, the condition can easily escape diagnosis or be misdiagnosed, which can cause severe consequences such as heart or kidney failure and sudden death. Therefore, early diagnosis is critically important.

Our patient was a child who was successfully diagnosed with TA, with the following clinical features: *Atypical clinical manifestation*. Our patient only had recurrent abdominal pain and vomiting, without fever or lameness. *Typical clinical signs*. Hypertension was found during physical examination, while low BP was detected in the extremities. Systolic vascular bruits could be heard. *Accelerated blood sedimentation*. This feature is consistent with the clinical diagnosis and is the result of enhanced CTA of the main arteries. All of the evidence mentioned above established our final diagnosis. The criteria of diagnosis in this case were based on the latest criteria developed by the Ankara conference in 2008, which includes the following standards: Imaging studies are essential for establishing the diagnosis of TA and for determining the extent of vascular involvement. In addition, a diagnosis can be established with at least one of the following: absent or weak peripheral impulses, discrepant BP between arms, arterial bruits, hypertension, and acute inflammatory symptoms including ESR > 20 mm/h or elevated CRP.^[4]^ Our patient met every criterion except that of showing impulse abnormality; therefore, the diagnosis could be made convincingly. Classification criteria have been developed for TA as a means of categorizing patients for research studies, and the most widely used set of criteria is one that was developed by Numano in 1996, which is based on imaging findings.^[5]^ In terms of our patient, the CTA results showing that the abdominal aorta and renal artery were involved suggest he should be classified as type IV. As mentioned above, imaging studies play a key role in the diagnosis of TA. Angiography has been considered the gold standard for diagnosing TA and serves as a complement to magnetic resonance angiography (MRA) or CTA, instead of being the first choice in clinical use. MRA, as the preferred choice in early diagnosis and long-term follow-up, can be sensitive to changes in the vascular wall but comparatively insensitive to changes in the distant branches of the arteries. CTA can detect early arterial lesions and can be more sensitive in evaluating disease activity than serologic tests; hence, CTA is more commonly used in diagnosing the middle or progressive stage of TA.^[6]^

The treatment of TA includes medication and surgery. The mainstay of therapy for TA is glucocorticoids. Approximately 60% to 80% of patients treated with glucocorticoids have been found to achieve symptom relief.^[7]^ Nevertheless, nearly half of these patients encounter recurrence in the process of glucocorticoid reduction.^[8]^ In the early stage of the disease, glucocorticoid is the medication routinely prescribed, and it can be combined with immunosuppressive agents as necessary; however, vascular intervention therapy is more important in the resting phase.^[9,10]^ Active antihypertensive and circulation improvement treatments are also needed to protect the vital organs. The purpose of these medications, including glucocorticoid, immunosuppressive agents, anticoagulation and antiplatelet agents, vasodilator drugs, antiinflammatory agents and traditional Chinese medicine, which can promote blood circulation, is to effectively suppress vascular inflammation, control BP, redress internal milieu disorder, and accelerate the development of the collateral circulation of the affected lesion to improve distal blood supply. Intervention therapy or surgery can be considered alternatives if arterial stenosis is found in the cervical, coronary or renal arteries or in the thoracic and thoracoabdominal aorta and if ischemic symptoms consequently develop; the aim of these treatments is to rebuild the focal blood flow.^[11]^ Intervention therapy or surgery cannot prevent disease development and can be provided only after the patient's condition is stable.

A multicentre study abroad with large samples has indicated that the low incidence and atypical symptoms of TA in children are responsible for its misdiagnosis in this population, which may lead to severe complications.^[12]^ Most patients have suffered from vascular stenosis for a long time, causing the impairment of multiple organs, such as cerebral infarction, aortic regurgitation, aneurysm, retinopathy, and heart failure. These complications are an important reason for the high mortality (35%, shown in a recent report^[13]^) of TA in children.^[10]^ The probable reason for the misdiagnosis of our patient was the lack of reported TA cases in children and the lack of specific clinical symptoms. The main complaint of our patient was abdominal pain, which, because an aneurysm is a common complication of TA, suggests that more detailed examinations should be given in the case of children with acute abdominal pain. Abdominal CT should be administered, peripheral impulses and arterial bruits should be checked, and BP among the 4 limbs should be monitored to rule out other plausible emergencies such as aneurysm caused by TA. Any patient with these suspicious symptoms should remain in the hospital for further examinations, such as angiography, CT, magnetic resonance imaging (MRI) or even digital subtraction angiography (DSA), if the condition permits. Above all, early diagnosis and treatment of TA play key roles in reducing complications and improving prognosis.

## Acknowledgment

We are grateful to all the clinicians involved in the management and treatment of the patient.

## Author contributions

**Investigation:** Shaobo Yang.

**Supervision:** Kuiran Dong.

**Writing – original draft:** Shaobo Yang.

**Writing – review & editing:** Shan Zheng.
